# Design of optimal solvent for extraction of bio–active ingredients from six varieties of *Medicago sativa*

**DOI:** 10.1186/1752-153X-6-123

**Published:** 2012-10-26

**Authors:** Angela Caunii, George Pribac, Ioana Grozea, Dorin Gaitin, Ionel Samfira

**Affiliations:** 1 Faculty of Pharmacy, University of Medicine and Pharmacy, “Victor Babes” 2A Eftimie Murgu Square, Timisoara, 300041, Romania; 2 Faculty of Medicine, Pharmacy and Dental Medicine, “Vasile Goldis” Western University, Arad, Romania; 3 Plant Protection Department, Grassland Department, Banat’s University of Agricultural Sciences and Veterinary Medicine from Timisoara, Calea Aradului no. 119, Timisoara, 300645, Romania

## Abstract

**Background:**

Extensive research has been performed worldwide and important evidences were collected to show the immense potential of plants used in various traditional therapeutic systems. The aim of this work is to investigate the different extracting solvents in terms of the influence of their polarity on the extracting ability of bioactive molecules (phenolic compounds) from the *M. sativa* flowers.

**Results:**

The total phenolic content of samples was determined using the Folin Ciocalteu (FC) procedure and their antioxidant activity was assayed through *in vitro* radical decomposing activity using the radical DPPH° assay (IUPAC name for DPPH is (phenyl)–(2,4,6–trinitrophenyl) iminoazanium). The results showed that water was better than methanol and acetic acid for extracting bioactive compounds, in particular for total phenolic compounds from the flowers of alfalfa. The average content of bioactive molecules in methanol extract was 263.5±1.02 mg GAE/100g of dry weight lyophilized extract. The total phenolic content of the tested plant extracts was highly correlated with the radical decomposing activity. However, all extracts were free–radical inhibitors, but the water extract was more potent than the acetic and the methanol ones. The order of inhibitor effectiveness (expressed by IC_50_) proved to be: water extract (0.924mg/mL) > acetic acid extract (0.154mg/mL) > methanol (0.079mg/mL). The profiles of each extract (*fingerprint*) were characterized by FT–MIR spectroscopy.

**Conclusions:**

The present study compares the fingerprint of different extracts of the *M. sativa* flowers, collected from the wild flora of Romania. The total phenolic content of the tested plant extracts was highly correlated with the radical decomposing activity. The dependence of the extract composition on the solvent polarity (acetic acid *vs.* methanol *vs.* water) was revealed by UV–VIS spectrometry and Infrared fingerprint.

## Background

The common name of the herb Alfalfa (*Medicago sativa*) is Lucerne. In folk medicine, this herb is used in alternative herbal treatments. The medicinal value of the plants lies in their phytochemical components which produce definite physiological actions in the organism. The most important bioactive components are starch, carbohydrates, basic proteins (histones and L–lysine, L–arginine, aspartic and glutamic acids) and the non–protein amino acid (L–canaverine). Alfalfa has high contents in tannins, pectin substances, saponines, amines, coumarin derivatives*,* triterpene glycosides, carotenoids*,* purines base, plant sterols*,* phytoestrogens (cumestrol)*,* flavones, isoflavonoids and phenolic compounds [1].

It is a remarkable source of vitamins A, D, E, and K. *M. sativa* belongs to the *Leguminosae* family; it is called the *"father of all plants*" and is considered the green food of the millennium. An important quality of alfalfa is the strengthening of the immunity. The ingredients of the alfalfa plant are used fresh, in order to maintain the essential nutrients necessary for proper functioning of the whole body [2-4].

The plant also contains a large amount of enzymes, anti-inflammatory substances, hormones, beta carotene, vitamin B_6_, vitamin C (four times more than citrus), and vitamin U, and contains trace minerals such as calcium, magnesium, iron, zinc, phosphorous and potassium and so on [5]. The leaves and young shoots of the plant show refreshing and uplifting quality. Alfalfa has a detoxifying effect on the body because of high percentage of water pectin (soluble fibres) enzymes, vitamins and minerals [6-8]. The vitamin U in alfalfa leaves prevents the injury to the gastric mucosal lining [9,10], assists in cell renewal and repairs the stomach and digestive system [11]. According to herbalists, it is effective in preventing water retention in the organism and is a popular tonic for convalescents when brewed into tea [12-14].

The leaves, seeds and sprouts of alfalfa have medicinal use in many metabolic deficiencies, are phytonutrient-rich, provide significant amounts of antioxidants [15-17], delay the aging processes, help to strengthen the immune system, especially protect against infection, prevent heart disease and coronary heart disease (through decreasing plasma cholesterol) [18,19]. Alfalfa contains numerous (hundreds) bioactive compounds, making it difficult to analyze and to ascribe healing properties of any particular component. In addition to the nutrients mentioned above, alfalfa contains two to three percent saponin glycosides and phenolic compounds. In our study, the extraction possibilities of the bioactive components of *M. sativa* are examined. There are many reports on biological activities of bioactive molecules, which could be relevant to the pharmacological effects. Different compounds may be present in different products depending on extraction methods [16]. For e.g., the alcoholic extracts stimulate bile excretion, whereas the aqueous extracts have no such effect [20-22]. Solvents differ in the extraction capabilities depending on their polarity and on the solute’s chemical structure. Solvents are selected according to the information available on the sample. The required extraction time varies depending on the sample; in some cases, solvent extraction occurs very quickly, in contrast to other cases when the materials must be allowed to mix [23] and sit for a while to achieve a proper extraction. The desired properties of solvents are a high distribution coefficient, good selectivity towards solute and little or no miscibility with feed solutions [24]. Usually, good solvents also exhibit some miscibility with feed solutions. Consequently, while extracting larger quantities of solute [25], the solvent could also extract significant amount of feed solution [26]. FTIR is a powerful tool for identifying types of chemical bonds in a molecule by infrared absorption spectrum which is a genuine molecular "fingerprint" (FT–MIR) [27].

In this study, the potential of FT-MIR spectroscopy is described in generating spectroscopic fingerprint of *M. sativa* flower extracts. The FT-MIR method allows the stability monitoring of the flower extracts and enables comparisons of selected extracts containing both identified and non-identified biocomponents [28].

## Results

**Figure 1:**
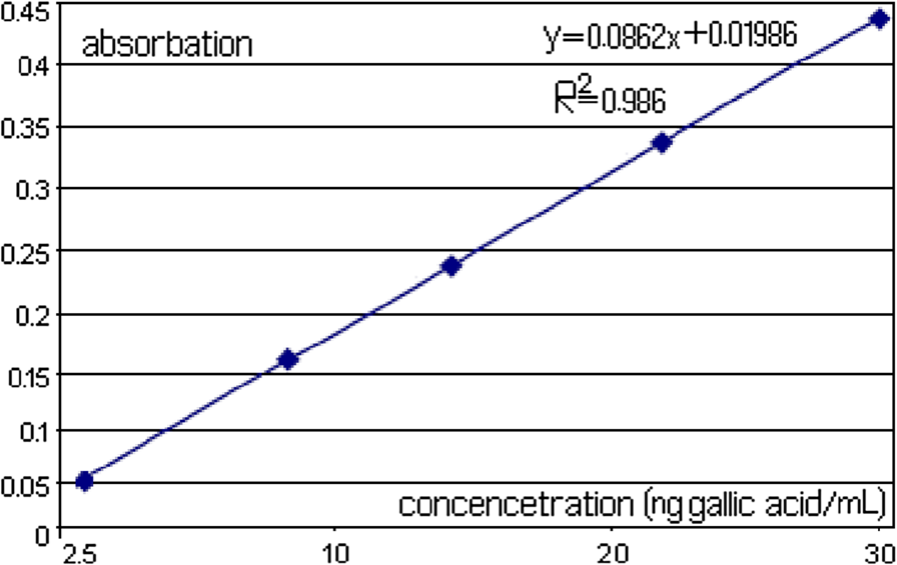
Standard curve.

The objective of the study was to screen the extracts resulting from *M. sativa* flowers for TPC, using different extraction solvents. The solvent polarity covers a wide range of dielectric constants: 33.1 for M, 6.2 for AA, and 78.57 for W. To evaluate the efficacy of various extraction techniques for phenolic compounds, GA was used a key compound. The calibration graphs used for analysis cover the range of 2.5–30ng/mL with r^2^ always greater than 0.98 (extraction yield (%), total phenolic content). Figure 1 shows a standard curve was plotted using gallic acid as a standard.

### Extraction factors of bioactive molecules, based on UV–VIS spectra

Comparative UV–VIS spectra of the AA, W and M extracts of the six plants were recorded, M being considered a “reference” solvent known to extract phenolic compounds and terpenoids from these plants. Based on their specific spectra, the mean values of extraction factors (EF) were calculated for each solvent (AA, W and M) from the absorbance values at λ_max_ for each plant extract (Table 1). For an integrated image of the differences between plants, solvent type and concentrations of bioactive molecules extracted, the EF mean values at 270–290 nm (for phenolic compounds derivatives extracted in AA, W and M) (EF_AA1_, EF_W1_, EF_M1_) and at 317–340 nm (for flavonoid derivatives) (EF_AA2_, EF_W2_, EF_M2_) were represented for each of the 6 samples. According to Table 1, it is evident that extraction factors in acidic M were superior to W and AA, especially for phenolic compounds (EF_AA1_, EF_W1_, EF_M1_) compared with flavonoid derivatives (EF_AA2_, EF_W2_, EF_M2_). Based on the differences of polarity between the three solvents used (M, W and AA), higher EF values have been noticed for samples 1, 3 and 4, richer in polar molecules, such as phenolic compounds. The differences in the extract yields from the tested plant materials might be assigned to different availability of extractable components, resulting from the varied chemical composition of plants.

**Table 1:** The absorption maxima of plants extract from UV–Vis spectra and the values EF

***M. sativa*flowers**	**λ**_**max.**_**(nm)**	**EFaa**	**EFm**	**EFw**
*M. sativa* (1)	279	7.68±0.06	221.4±0.03	240.0±0.06
	320	7.86±0.08	201.4±0.01	280.0±0.03
	396	2.07±0.02	106.8±0.02	142.6±0.02
	652	83.08±0.08	43.86±0.01	123.7±0.01
*M. sativa* (2)	279	82.84±0.08	41.18±0.06	81.71±0.01
	320	87.06±0.02	42.86±0.06	73.31±0.02
	396	82.19±0.03	41.21±0.01	66.03±0.01
	652	72.11±0.01	223.3±0.04	242.0±0.06
*M. sativa* (3)	279	67.78±0.06	103.6±0.01	238.0±0.01
	320	48.84±0.06	72.86±0.00	218.0±0.03
	396	13.14±0.01	42.86±0.08	62.0±0.03
	652	12.11±0.02	32.16±0.01	8.81±0.02
*M. sativa* (4)	279	67.81±0.01	127.8±0.02	222.0±0.06
	320	52.42±0.01	121.1±0.05	218.0±0.08
	396	33.88±0.03	117.1±0.02	142.0±0.02
	652	18.03±0.01	82.68±0.08	108.0±0.01
*M. sativa* (5)	279	28.76±0.06	37.88±0.08	142.0±0.08
	320	26.13±0.01	38.26±0.07	97.0±0.01
	396	18.76±0.02	78.01±0.08	72.0±0.04
	652	13.16±0.09	19.13±0.02	53.1±0.02
*M. sativa* (6)	279	31.82±0.06	26.81±0.08	102.0±0.03
	320	28.84±0.01	24.86±0.06	68.4±0.0„
	396	22.23±0.20	22.38±0.04	52.27±0.00
	652	12.35±0.01	11.31±0.03	22.68±0.04
TPC (mg GAE/g extract) ± SD		197.9±0.03	263.5±1.02	167.3±3.02
IC_50_ mg/mL		0.079±0.00064	0.924±0.01188	0.154±0.00129

Values (mean ± SD) are average of three samples of each *M.sativa* material, analyzed individually in triplicate (*n*=1x3x3); Values extraction factors(EF)=mean of three values calculated (X±SD).

### Total phenol content (TPC)

The average TPC of the extraction from alfalfa flowers, tested for each solvent type, were presented in Table 1. The phenolic compounds extracts of plants are always a mixture of different classes of phenols selectively soluble in the solvents. Methanol is the best solvents for extraction of phenolic compounds from alfalfa flowers. Water is an inefficient solvent for the extraction of TPC from the *M. sativa* flowers studied.

### Antioxidant activity: DPPH assay

**Figure 2:**
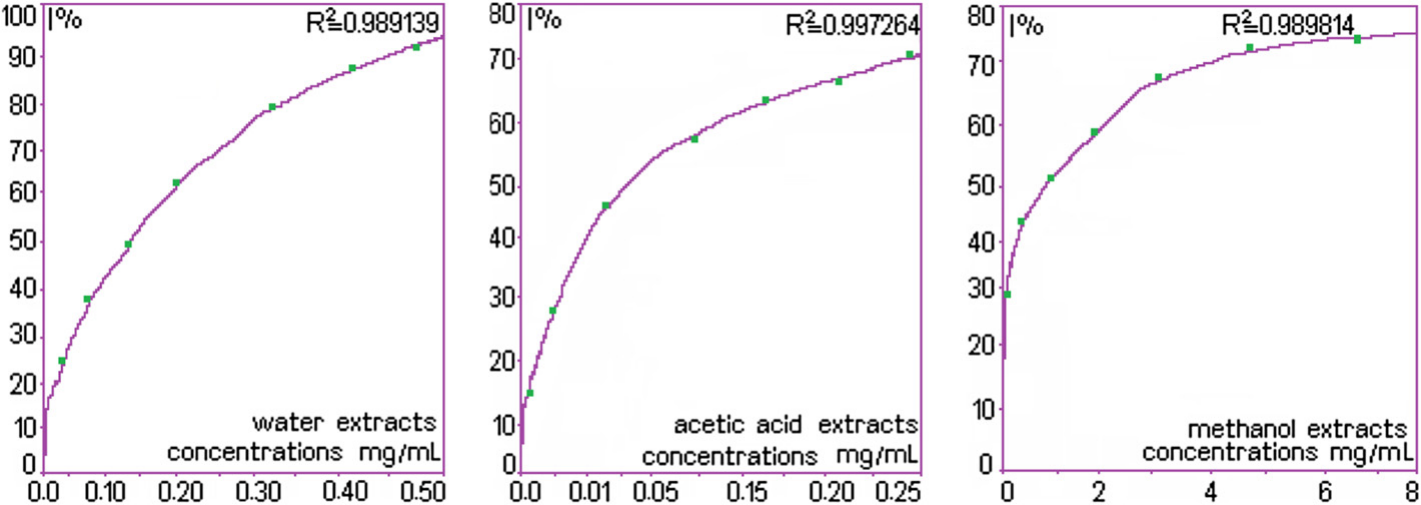
**Decomposing capacity of four *Medicago sativa *flowers extracts expressed in percentage at different concentrations (acetic acid extract; methanol extract; distilled water extract).**

The stable radical DPPH° has been widely used for screening of substances with potential antioxidant activity measured by the decolorizing effect as a result of trapping the impaired electrons of DPPH°. Lower values of IC_50_ indicate higher antioxidant activity (Figure 2). Every extract presented a good decomposing activity, but using W, AA and M as solvent displayed a powerful antioxidant activity. These activities in the following decreasing order were: AA extract (0.079mg/mL±0.00064)> W extract (0.154mg/mL±0.00129)>M extract (0.924mg/mL ± 0.01188). The extract of the alfalfa flowers, obtained with W has presented a strong and potent decomposing capacity against free radical DPPH°, whereas with the same solvent, we recorded the lowest polyphenols compared to other solvents obtained with the FC method.

### FT–MIR fingerprint

**Figure 3:**
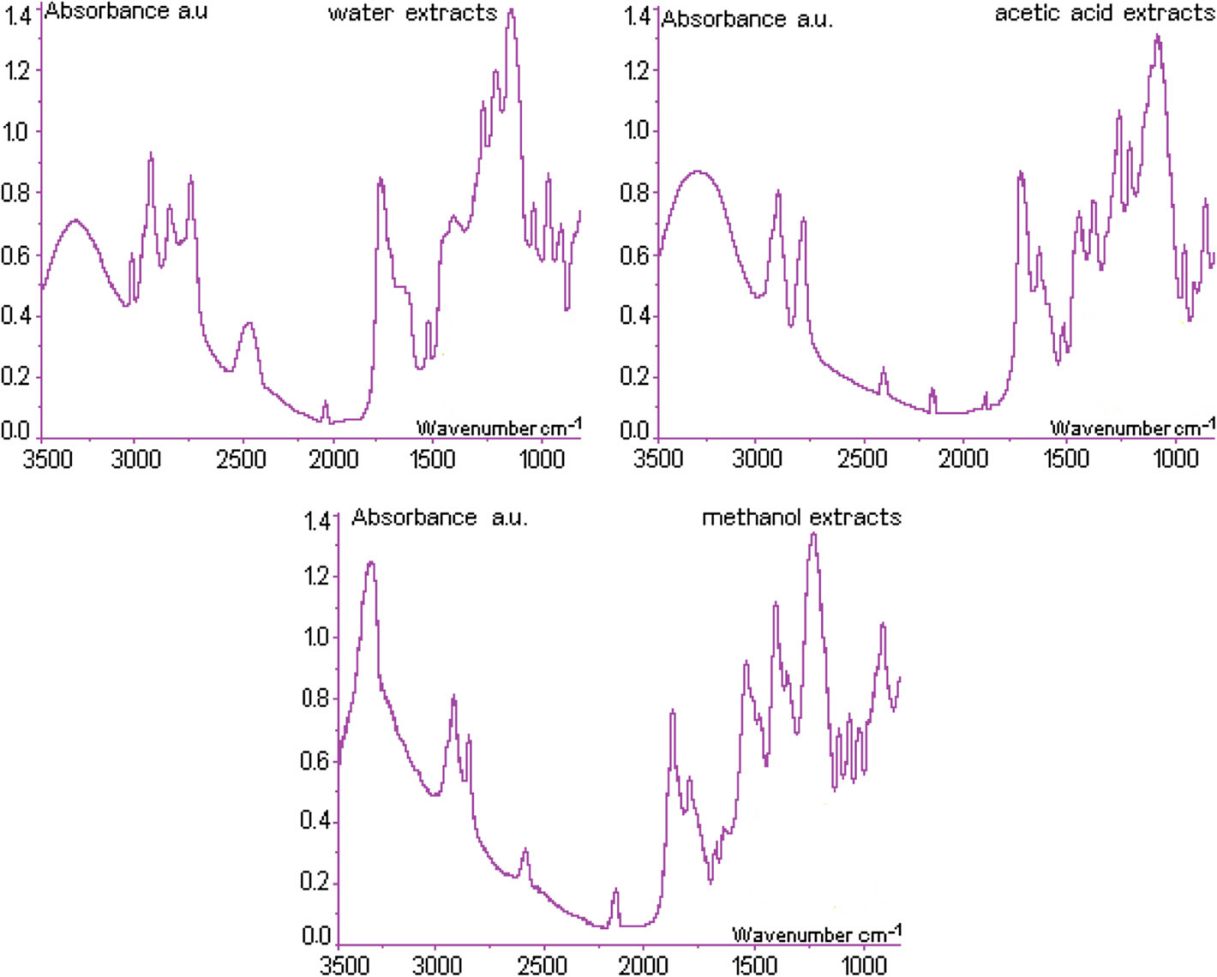
**The FTIR fingerprint of the extracts of the studied plants *M. sativa *(distilled water extract; acetic acid extract; methanol extract).**

The FT–MIR spectra (4000–900 cm^–1^) of AA and W extracts of each plant were registered and the specific wavenumbers and intensities were considered. Figure 3 presents the FT–MIR spectra of methanol extracts and Table 2 show the corresponding absorption peak area for specific regions. Table 2 and 3 include the biocomponents in methanol extracts determined by FTIR and by spectrometry. The functional groups identification was based on the FTIR bands attributed to stretching and bending vibrations.

**Table 2:** **Cumulative data–identification of Raman marker bands for the investigated extracts of*M. sativa***

**Vibration mode (cm**^**–1**^**)**		
stretching	bending	
ϑ(C=C)	δ(=C–H)	δ(–C–H)
1657 s	1301 m	1264 w
1657 s	1302 m	1265 m
1657vs	1302 m	1264 m
1657 s	1302 m	1265 m
1657 vs	1302 m	1265 ms
1657 s	1302 m	1265 m

**Table 3:** **The typical infrared absorption peak areas for specific regions for the investigated extracts of*M. sativa***

**No **Group frequency, wavenumber (cm**^**–1**^**)/Assignment****	**Functional Class**
1 <1000 cm^–1^ [*750–720 Methylene–(CH*_*2*_*)n–rocking, 970–960 trans-C–H out-of-plane bend; 700 (broad) cis–C–H out-of-plane bend*]	Isoprenoids
2 997–1130 cm^–1^ [*1050–990 Aliphatic phosphates (P–O–C stretch)*]	mono–, oligo– carbohydrates
3 1150–1270 cm^–1^ [*1210–1150 Tertiary amine, CN stretch*]	acid or ester
4 1300–1450 cm^–1^ [*1350–1260 Primary or secondary, OH, in-plane bend 1410–1310 Phenol or tertiary alcohol, OH bend*]	Amide, phenyl groups
5 1500–1600 cm^–1^ [*1610–1550/1420–1300 Carboxylate (carboxylic acid salt); 1680–1630 Amide*]	amino acids
6 1600–1760 cm^–1^ [*1615–1580 Aromatic ring stretch*]	Aldehydes, cetones, esters
7 2800–2900 cm^–1^ [*2970–2950/2880–2860 Methyl C–H asym./sym. Stretch*]	lipids, metoxy derivatives (*cis* double bonds)
8 3000–3600 cm^–1^ [*3095–3075 Terminal (vinyl) C–H stretch; 3040–3010 3095–3075 vinylidene C–H; Stretch 3040–3010 Medial, cis–or trans–C–H; Stretch, 3570–3200 (broad) Hydroxy group, H-bonded; OH stretch 3400–3200 Normal polymeric OH Stretch 3550–3450 Dimeric OH stretch; 3570–3540 Internally bonded OH stretch*]	water, alcohols, phenols, carbohydrates, peroxides

## Discussion

Samples 1 and 3 had similar EF in M and W, sample 2 was better extracted in AA and was richer in phenolic compounds derivatives [29]. The components of sample 4 were extracted two times better in M than in W, and low EF values in AA is an indication of polar active molecules [30]. Sample 5 and 6 contained reduced concentrations of phenolic compounds, but exhibit high absorptions in methanol at 279 and 320 nm, respectively, which might be attributed to higher concentrations of lignans and terpenoids [31]. Of therapeutic reasons, it has been considered that AA extracts or M extracts can provide higher concentrations of bioactive molecules from these plants.

The average TPC (mg GAE/g crude extract) of the water methanol extract was significantly higher (263.5 mg/g) than that for methanol, (167.3 mg/g) and better than that for acetic acid extracts (197.9 mg/g). The use of water presents the advantage of modulating the polarity of alcoholic solvents. The solubility of polyphenols depends mainly on the hydroxyl groups, the molecular size and the length of the hydrocarbon chain [32,33]. Another remarkable observation refers to the higher yield of extract related to solvent M, followed by water as solvent. Water is an inefficient solvent of the extraction of TPC from the *M. sativa* flowers studied [32,33]. The average TPC (mg GAE/g crude extract) of the methanol extract was significantly higher (263.5 mg/g) than that of W, (167.3 mg/g) and better than that for AA extracts (197.9 mg/g).

The solubility of polyphenols depends mainly on the hydroxyl groups, the molecular size and the length of the hydrocarbon chain. Another remarkable observation refers to the higher yield of extract related to solvent M, followed by water as solvent.

The details in Table 1 explain the higher total phenolic compounds when we choose organic solvents whose polarity is modified with water. These mixtures become ideal and selective to extract a great number of bioactive compounds of phenolic type.

Whereas methanol offers a higher amount of yield, it is not appropriate to extract polyphenols. The solvent extracts only the water-soluble bioactive compounds; moreover, many other residual substances/impurities are present in the extracts.

It appears from our results that some of phenolic compounds and other pharmacologically interesting compounds from the samples are not extractible with plain water, for this reason the mixtures of solvents are suitable to extract different bioactive compounds. In our investigation, the mixture of methanol and water proved to a better solvent for the extraction of phenolic compounds from plants flowers than the mixture of AA and water. On the other hand, the M extract has higher total phenolic compounds content than AA, and W extracts, but did not exhibit the highest antioxidant activity among the three different extracts. In this context, it is possible that phenolic compounds, existing in the water extract, possess an ideal structure for decomposing free radicals since they possess a number of hydroxyl groups acting as hydrogen donors turning them into important and very powerful antioxidant agents.

The results of this accounts for the reason why for each solvent, taken individually, the TPC determined with the FC assay presents a good correlation with antioxidant activity, but it is not the case when compare between extracts obtained by various solvents. Different reports are found in the literature: whereas some authors have found a correlation between the total phenolic compounds content and the antioxidant activity, others found no such relationship [33].

Antioxidant activity of extracts is strongly dependent on the solvent due to the different antioxidant potentials of compounds with different polarity. The FC assay offers an estimate of the TPC present in an extract. The assay is not specific for polyphenols; instead many interfering compounds may react with the reagent resulting in apparently elevated phenolic compounds concentrations.

In addition, various phenolic compounds respond differently in this assay, depending on the number of their phenolic groups and the TPC does not incorporate necessarily all the antioxidants that may be present in an extracting.

According to this study, methanol appears ideal for extracting a high amount of phenolic compounds, while water was the ideal solvent for extract bioactive compounds from *M. sativa* flowers with potential antioxidant activity content.

Eight areas were identified as the MIR domain and the fingerprint region was localized between 900 and 1500 cm^–1^[32]. Absorptions below 1000 cm^–1^ correspond to C–H bending vibrations from isoprenoids, the absorption bands between 997–1130 cm^–1^ may be attributed to stretching vibrations C–O of mono–, oligo– and carbohydrates, with signals at 1030, 1054, 1104, and 1130 cm^–1^, while the absorption over the range of 1150–1270 cm^–1^ corresponds of stretching vibrations of C–O fragment of carbonyl group or to O–H bending vibration. Absorption situated between 1300–1450 cm^–1^ corresponds to stretching vibrations C–O (amide) and C–C stretching vibration of the phenyl groups, while the signals between 1500–1600 cm^–1^ may be assigned to aromatic parts and to N–H bending vibrations. Between 1600–1760 cm^–1^ there is a complex corresponding to bending vibrations N–H (amino acids), C=O stretching vibrations (aldehydes, ketones and esters) as well as to free fatty acids (1710 cm^–1^) and glycerides (1740 cm^–1^) [32]. The absorption comprised in domain 2800–2900 cm^–1^, corresponds to C–H stretching vibrations, specific to CH_3_ and CH_2_ in lipids, methoxy derivatives and to C–H in aldehydes, including *cis* double bond configuration. The domain 3350–3600 cm^–1^ corresponds to stretching vibrations of OH groups (water, alcohols, phenols, carbohydrates, peroxides) as well as to amides (3650 cm^–1^). In methanol extracts there are absorption bands in the 1300–1800 cm^–1^ domain, more than in W, e.g. at 1558, 1517 and 1467 cm^–1^, as well as in the region 1380–1450 cm^–1^. Such differences were noticed also by other authors, after processing the second derivative in *M. sativa* flowers extracts, where typical signals, specific to cellulose and hemicelluloses at 3413 and 1054 cm^–1^, were found.

The signals at 1642 and 1536 cm^–1^ correspond to the amide I band (carbonyl group) and amide II (stretching ϑCN + bending ϑNH) found in glycoproteins [33].

Carbonyl groups have specific signals at 1743 cm^–1^. Due to observation of region 1 (specific to terpenoids), it has been noticed that samples 6, 5 and 4 possess bands located at higher wavenumbers in AA, similarly to the results of UV–spectra.

In the other IR regions (4 and 6) no significant differences between the three solvent extracts were noticed, but in regions 2 (corresponding to glucosides) and 7 (lipids), in all plant extracts, the M extract was significantly more charged in molecules than AA or W extracts. Finally, the phenolic compounds concentrations determined by the FTIR method, based on the peak intensity at 1743 cm^–1^, and total phenolic compounds content calculated using the VIS spectrometry have been compared.

A significant (p<0.05) correlation factor was obtained; it is known that the measurement performed within the VIS spectrometry is not specific to phenols and can overestimate concentrations, while the FTIR method, using the absorption bands (950–1900 cm^–1^) estimation can also lead to false results.

It can be considered in this case that measurements, based on the FTIR absorption intensity at 1743 cm^–1^, offer the best evaluation of the concentration of phenolic compounds in these plants. This work has been undertaken to gain an understanding of the chemical composition of latent prints so that new methods of developing fingerprint images can be explored. Additionally, methods of imaging fingerprints from electro-optical responses obtained through spectrometers have been investigated.

## Conclusions

The data of study showed that UV–VIS spectrometry and FT–MIR spectroscopy are adequate techniques for comparative fingerprinting and for evaluate the extraction yield of folk herbs. Based on UV spectrometry, the extraction yields were superior in acidic M in comparison to W and AA, ensure increased yield in phenolic compounds comparative to flavonoid derivatives. Due to the differences of polarity between the three solvents used, higher extraction yields were obtained for *M. sativa*, sample (2) sample (3) and (4) sample, richer in phenolic compounds. Samples 5 and 6 had lower concentrations of phenolic components, but higher content of lignans and terpenoids.

Based on FT–MIR spectroscopy, for each plant extract, the fingerprint region was determined, located between 900 and 1500 cm^–1^ and the specific functional groups involved have been identified. Every FTIR data will be correlated and further validated in comparison to the detailed HPLC analysis of the same extracts, in order to validate the FTIR method as an optimal tool to investigate the fingerprint and to predict the composition of plants or to evaluate the quality and authenticity of different standardized formulas. In any phytotherapeutic research, it is necessary to choose solvent according to biological activity required and not pursuant to that providing a high amount of bioactive compounds. Thus, the methanol extract or fraction, expressing good biological capacity, indicates that the substance with powerful biological effect exists in this extract and it have to be isolated and purified to confirm its pharmacological and medical use.

The outcomes showed that this approach can be used to monitor the composition of extracts, allowing to monitor chemical changes that may occur during storage periods and to investigate the occurrence of a determined biocomponent in different extracts.

The FDA specifies the extracts can be stored indefinitely in a sealed airtight container kept in a cool dark place. Do not refrigerate *M. sativa* flowers extract.

The water extract appeared to have good antioxidant activities. Further investigations are necessary to verify these activities in vivo.

## Methods

### Extraction of the *M. sativa* flower

Alfalfa flowers were collected from a certified farm (Banat region, western Romania) during early summer. The six varieties of *M. sativa*, was collected in different locations. Flowers were separated manually from aerial parts and washed with tap water prior to freeze–drying. Voucher samples are archived in the laboratory and are available for analysis by contacting the corresponding author.

Aliquots of 20 g from each dried and grounded plant (selected from 100 g mix of the flowers) were extracted in 85 mL solvent consisting of methanol 90% in water acidulated by 1% hydrochloric acid (M), or acetic acid (AA), or distilled water (W).

After 30 min sonication, centrifugation and filtration, the clear extracts were kept in the deep freezer until analysis [25].

### UV–VIS spectra and calculation of extraction factors

The UV–VIS spectra (700–200 nm) were recorded for each extract (AA, W or M) using PG spectrometer Instruments UV–VIS the specific soft of instrument, UV WIN 5.05. The wavelengths of specific absorption maxima of phenolic compounds (280 and 330 nm), carotenoids (420–470nm) and/or chlorophylls (663 nm) were identified.

In order to compare the yields of extraction in different solvents, the extraction factor (EF) of bioactive molecules from each extract has been calculated, considering the absorption values (A at λ_max_) recorded for each λ_max_, multiplied with the dilution factor (d) and applying the relation: **EF=A(λ**_**max**_**)⋅d**.

The results were expressed as mean values of four samples per plant and in triplicate extracts from each plant. The content of phenolic compounds was determined by spectrometry, using the standard FC method [33,34].

### Total phenol content (TPC)

TPC of the various alfalfa flowers extracts AA_E_, W_E_ and M_E_ was estimated by spectrometric assay, using a FC reagent [33]. The absorbance of developed pigment was determined at 725nm. Briefly, for each extract, 1mL of extract dissolved in methanol was mixed with 7.5mL FC reagent (diluted 10 fold), the mixture kept at 22°C for 5 min, then a volume of 7.5mL Na_2_CO_3_ solution (60g/L) was added. The absorbance was read after 90min. TPC values were determined using a standard curve prepared with Gallic acid (GA). Results were expressed as mg GA Equivalent (GAE) per 100g dry weight of lyophilized extract. The TPC was carried out in triplicate.

### Antioxidant activity (DPPH assay)

All lyophilized extracts were dissolved in methanol. The antioxidant capacity was determined by DPPH°. The DPPH° solution was prepared by dissolving (phenyl)–(2,4,6–trinitrophenyl) iminoazanium in methanol to 6x10^–5^M concentration. 3.9mL M DPPH solution was added to each 0.1 mL extract obtained with different solvents. The absorbances were read after 30 min at 515 nm. The inhibition activity percentage was calculated according to the relation **{[(A**_**c**_**–A**_**t**_**)/A**_**c**_**]⋅100}**, where A_c_ stands for the absorbance of the control and A_t_ is the absorbance of the extract. The inhibition curves were plotted and the IC_50_ values, defined as the amount of antioxidant necessary to decrease the initial DPPH° concentration by 50%, were determined.

### FT–MIR measurements

The Fourier Transform Infrared Spectrum (FTIR) of each extract was recorded in the optical region, from 4000 to 900 cm^–1^. In order to improve the signal to noise ratio, 64 scans were accumulated in each spectrum recording. Horizontal Attenuated Total Reflection (HATR) device and an IR-Press of the FTIR spectrometer (JASCO 660 PLUS) were used. The spectral data were processed with the IR solution Software Overview and OriginR 7SR1 Software. The spectra were registered both as fluid (AA, W and M) and as evaporated extracts (these latter data being not shown). TPC were determined also by FTIR method, either using the band intensity at 1742 cm^–1^ or from the area between the region 950–1900 cm^–1^, with reference to the calibration curve obtained with pure GAE (range of concentrations 2.5–30 ng/mL M) [35].

### Statistical analysis

Values were expressed as mean ± S.D. Statistical significance was evaluated by Students–"t" test at 5% level of significance (p < 0.05).

## Abbreviations

AA: Acetic Acid; M: Methanol; W: Distilled Water; GA: Gallic Acid; DPPH°: Radical (phenyl)–(2,4,6–trinitrophenyl) iminoazanium; FC reagent: Folin Ciocalteu reagent; TPC: Total Phenolic Compounds; EF: Extraction Factor.

## Competing interests

The authors declare that they have no competing interests.

## Authors' contributions

These authors contributed equally to this work. All authors read and approved the final manuscript.
